# Implementation of positron emission tomography radiopharmaceuticals labeled with Gallium-68 in a hospital radiopharmacy: experience with more than 7,500 patients

**DOI:** 10.31744/einstein_journal/2025AE1552

**Published:** 2025-11-24

**Authors:** Lilian Yuri Itaya Yamaga, Marycel Rosa Felisa Figols de Barboza, Luciana Malavolta, Leonardo Lima Fuscaldi, Jorge Mejía Cabeza, Solange Amorim Nogueira, Gilberto Szarf, Marcelo Livorsi da Cunha, Taise Vitor, Guilherme de Carvalho Campos, Jairo Wagner, Marcos Roberto Gomes de Queiroz

**Affiliations:** 1 Hospital Israelita Albert Einstein São Paulo SP Brazil Hospital Israelita Albert Einstein, São Paulo, SP, Brazil.; 2 Faculdade de Ciências Médicas da Santa Casa de São Paulo São Paulo SP Brazil Faculdade de Ciências Médicas da Santa Casa de São Paulo, São Paulo, SP, Brazil.

**Keywords:** Positron-emission tomography, Gallium-68, Radiopharmaceuticals, Radionuclide generators

## Abstract

In this study, we report the implementation of in-house synthesis of gallium-68 (^68^Ga)-labeled radiopharmaceuticals for positron emission tomography (PET) at a tertiary hospital radiopharmacy. [^68^Ga]Ga-DOTATATE and [^68^Ga]Ga-PSMA-11 were introduced into clinical practice in January 2014 and October 2015, respectively, following the establishment of routine automatic module synthesis under good manufacturing practice (GMP) and a certified ^68^Ge/^68^Ga generator. Quality control tests, including radiochemical yield, purity, and pyrogenicity, demonstrated high labeling efficiency, excellent purity, and absence of pyrogens. Between January 2014 and October 2024, our radiopharmacy performed 755 [^68^Ga]Ga-DOTATATE and 2,429 [^68^Ga]Ga-PSMA-11 syntheses, enabling PET scans for 1,636 and 5,892 patients, primarily for the evaluation of neuroendocrine tumors and prostate cancer, respectively. Two new PET tracers, [^68^Ga]Ga-DOTA-Ubiquicidin^[29-41]^ (for infection imaging) and [^68^Ga]Ga-FAPi-46 (targeting cancer-associated fibroblasts), were introduced in December 2021 and December 2023, respectively. Both demonstrated high radiolabeling yields and purities. After preclinical studies and ethics approval, PET scans using these tracers produced high-quality images with favorable preliminary clinical outcomes. To the best of our knowledge, our institution is among the first in Brazil to achieve in-house production and clinical application of these two novel tracers. Over the past decade, the automated GMP-compliant system has consistently enabled reliable synthesis of ^68^Ga-labeled radiopharmaceuticals, ensuring high radiochemical yield, purity, and high-quality PET imaging, thereby expanding clinical practice with safe and effective new agents.

## In Brief

This study reports the decade of in-house synthesis of 68Galabeled radiopharmaceuticals ([68Ga]Ga-DOTATATE and [68Ga] Ga-PSMA-11) for PET imaging under GMP at a tertiary hospital, enabling over 7,500 PET scans. Novel tracers, [68Ga]Ga-DOTAUBI and [68Ga]Ga-FAPi-46, were successfully introduced and synthesized with high yield and purity, and were used to obtain high-quality clinical images.

## Highlights

■ In-house 68Ga radiopharmaceutical synthesis under GMP in a tertiary hospital radiopharmacy.
■ Over 7,500 patients underwent imaging with [68Ga]Ga-DOTATATE and [68Ga]Ga-PSMA-11.
■ This is the first radiopharmacy in Brazil to introduce [68Ga] Ga-DOTA-UBI, and [68Ga]Ga-FAPi-46.

## Graphical abstract

**Figure:**
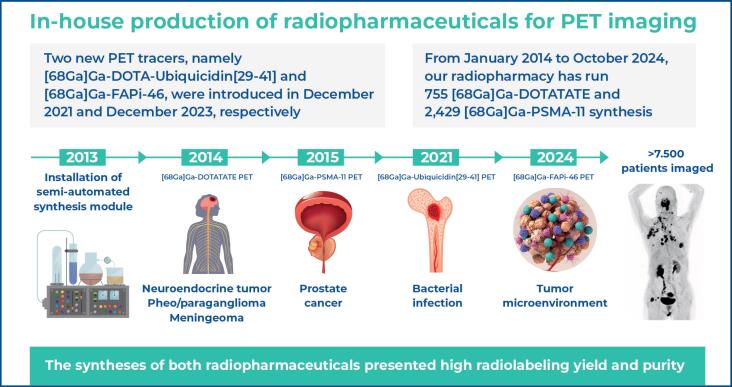

## INTRODUCTION

Positron emission tomography (PET) is a well-established imaging modality for the *in vivo* evaluation of numerous biological processes. It provides clinically important information for tumor diagnosis and staging, differentiation between aseptic inflammation and infection, and assessment of neurological diseases. PET enables early detection of functional abnormalities that precede the morphological changes typically observed in conventional cross-sectional imaging, such as computed tomography (CT) and magnetic resonance (MR) imaging. The number of PET radiopharmaceuticals used in nuclear medicine has steadily increased in recent years, with expanding applications across many clinical conditions.^(1)^

[¹^8^F]Fluorodeoxyglucose ([¹^8^F]FDG) remains the most widely used PET radiopharmaceutical worldwide and is regarded as the standard tracer for oncologic imaging. [^18^F]FDG is a glucose analog labeled with the positron-emitting radioisotope fluorine-18 (^18^F). Its production, however, requires a cyclotron facility, which entails high infrastructure and operational costs as well as specialized technical support.^(2)^

In this context, the generator-based production of positron-emitting isotopes offers an important alternative for PET imaging. One such isotope is gallium-68 (^68^Ga), a positron emitter with a physical half-life of 68 min. The main advantages of ^68^Ga over ¹^8^F include its generator availability, independence from a cyclotron, versatility in radiopharmaceutical development, and compatibility with fully automated synthesis modules.^(3)^

Another limitation of [^18^F]FDG PET is its reduced sensitivity for detecting slowly growing tumors, such as prostate carcinoma and neuroendocrine tumors (NETs), which may show little or no [^18^F]FDG uptake. Additionally, [¹^8^F]FDG accumulation at sites of inflammation can hinder differentiation between malignant and inflammatory or infectious processes.^(2)^

To address these challenges, ^68^Ga-labeled radiopharmaceuticals, including DOTA-modified peptides and prostate-specific membrane antigen (PSMA) inhibitors, have been successfully introduced into clinical practice as alternatives to [^18^F]FDG. The development of somatostatin analog peptides – namely DOTA-TOC, DOTA-NOC, and DOTA-TATE – represented a major advance in ^68^Ga radiopharmaceuticals and provided the foundation for subsequent tracers (Table 1).^(4-6)^

**Table 1:** Mechanisms of uptake and main clinical indications of [^68^Ga]Ga-DOTATATE, [^68^Ga]Ga-PSMA-11, [^68^Ga]Ga-DOTA-Ubiquicidin_[29-41]_, and [^68^Ga]Ga-FAPI-46

Radiopharmaceutical	Mechanism of uptake	Clinical Indications	
[^68^Ga]Ga-DOTATATE	High affinity to SSTR overexpressed in the cell membrane of many tumor types	NET	
			Initial staging after histological diagnosis of NET
			Localization of primary tumor in patients with known metastatic disease but unknown primary
			Selection of patients for SSTR-targeted PRRT ([^177^Lu]Lu-DOTATATE)
			Restaging at time of suspected NET progression
		Pheochromocytoma/Paraganglioma	
			Tumor characterization and therapeutic decision making
		Neuroblastoma	
			Diagnosis and evaluation of disease extent, especially in negative or inconclusive [^123^I]I-metaiodobenzylguanidine scintigraphic findings
			Suitability for PRRT therapy ([^177^Lu]Lu-PSMA)
	
[^68^Ga]Ga-PSMA-11	Binding to PSMA, a transmembrane glycoprotein that is overexpressed in PCa	Localization of PCa in the setting of biochemical recurrence	
		Primary staging in high-risk disease before surgical procedures	
		Staging before [^177^Lu]Lu-PSMA radioligand therapy to confirm target-expression	
		Directed biopsy after previous negative biopsy in patients with high suspicion of PCa	
[^68^Ga]Ga- DOTA-Ubiquicidin_[29-41]_	Fragment of a peptide that binds to the bacterial cell membrane	Chronic osteomyelitis	
		Fever of unknown origin	
		Suspected prosthesis infection	
[^68^Ga]Ga-FAPi-46	Binding to FAP, a molecular target which is highly expressed in the stroma of epithelial tumors.	Emerging clinical applications: detection of primary tumors, nodal and distant metastases, particularly of tumor types with decreased [^18^F]FDG uptake (hepatocellular, gastric, pancreatic, lung and breast carcinomas, sarcoma etc.). Rheumatoid arthritis, Crohn disease, fibrosis (liver, kidney, lung).	

NET: neuroendocrine tumors, SSTR: somatostatin receptor, PRRT: peptide receptor radiation therapy, PSMA: prostate-specific membrane antigen, PCa: prostate cancer, FAP: fibroblast activation protein, FAPi: fibroblast activation protein inhibitor.

The increasing demand for routine clinical production of ^68^Ga-compounds has been efficiently met with the development of automated systems, which provide robust, fast, and reliable radiopharmaceutical synthesis with low running and maintenance costs. ^(7,8)^

This article presents our 10-year experience in implementing the in-house synthesis of [^68^Ga]Ga-DOTATATE and [^68^Ga]Ga-PSMA-11 for PET imaging in a hospital radiopharmacy from January 2014 to October 2024. The introduction of new ^68^Ga-labeled tracers, such as [^68^Ga]Ga-DOTA-Ubiquicidin^[29-41]^ and [^68^Ga]Ga-FAPi-46, is also described.

### Implementation of the automated production process of ^68^Ga-labeled radiopharmaceuticals

Automated synthesis systems provide reliable, reproducible, and safe production of radiopharmaceuticals for clinical use. They also reduce radiation exposure to technical staff, minimize operational errors, and have low maintenance requirements and acceptable operating costs.^(8)^

A Modular-Lab Standard synthesis module (Eckert & Ziegler, Berlin, Germany) was successfully installed and implemented in November 2013 in the hospital radiopharmacy department of our nuclear medicine service, which is part of the Imaging Department. This was the first nuclear medicine service in Brazil to implement an automated synthesis system. ^68^Ga was obtained using an IGG 100 ^68^Ge/^68^Ga generator (Eckert and Ziegler).

A quality control system was implemented to ensure high quality and safety for the synthesis of [^68^Ga]Ga-DOTATATE, [^68^Ga]Ga**-**PSMA-11, [^68^Ga]Ga-DOTA-Ubiquicidin_[29-41]_ and [^68^Ga]Ga-FAPi-46. Quality control parameters included:

1. Radiochemical yield and purity, using high-performance liquid chromatography (HPLC, 1290 Infinity II, Agilent Technologies, Santa Clara, CA, USA) and thin-layer chromatography (TLC) scanner (AR-200, Eckert & Ziegler, Valencia, CA, USA).
2. Radionuclidic purity, using a gamma counter (Wizard 2TM 3" 2480, Perkin Elmer, USA).
3. Pyrogen evaluation (Endosafe Nexgen-PTS, Charles River Laboratories, Wilmington, MA, USA).
4. pH testing.

The synthesis and quality control of ^68^Ga-labeled tracers typically take less than 30 min, providing fast and highly reproducible processes suitable for routine PET imaging, in accordance with national health regulatory standards (Resolution RDC n^o^ 63, 2009, *Agência Nacional de Vigilância Sanitária – ANVISA*) and the European Pharmacopeia monograph.^(4)^

Over 10 years, the automated module conducted clinical synthesis of [^68^Ga]Ga-DOTATATE, [^68^Ga]Ga-PSMA-11, [^68^Ga]Ga-DOTA-Ubiquicidin_[29-41_], and [^68^Ga]Ga-FAPi-46 under GMP standards using certified reagents and consumables.

### [^68^Ga]Ga-DOTATATE

[^68^Ga]Ga-DOTATATE is a somatostatin analog peptide that binds to somatostatin receptors (SSTR) overexpressed in neoplasms, particularly gastro-entero-pancreatic neuroendocrine tumors (NETs). Other tumors, such as pheochromocytoma/paraganglioma, neuroblastoma, and meningioma, also overexpress SSTRs and can be detected using [^68^Ga]Ga-DOTATATE PET.^(9-12)^

DOTATOC and DOTANOC are other SSTR-targeting peptides with similar binding affinities. ^68^Ga-labeled peptide PET imaging is considered the gold standard for detection and initial staging of NETs and is also useful for selecting patients with metastatic NET for peptide receptor radionuclide therapy (PRRT) with [^177^Lu]Lu-DOTATATE.^(13,14)^

Other clinical indications for [^68^Ga]Ga-DOTATATE PET are summarized in table 1.

### [^68^Ga]Ga-DOTATATE synthesis and quality control

After training on the automated synthesis module and standardization of quality control methods, the production process for [^68^Ga]Ga-DOTATATE was established using GMP-grade DOTATATE acetate as the precursor and synthesis cassettes supplied by ABX Advanced Biochemical Compounds (Radeberg, Germany). The methodology has been previously described in detail.^(15,16)^

In summary, [^68^Ga]GaCl^3^ was purified using a cation-exchange resin to remove metallic impurities and residual ^68^Ge. The purified [^68^Ga]GaCl^3^ was added to reaction vials containing 40μg of DOTATATE dissolved in 2mL of 0.1 M sodium acetate buffer (pH 4.0). Radiolabeling was performed at 85°C for 5 min. The product was purified using a Sep-Pak C18 cartridge and sterilized by filtration through a 0.22-µm Millipore membrane filter.

Radiochemical yield was calculated as the percentage of total activity recovered in the product vial relative to total activity, including residual activity in the synthesis module. Radiochemical purity was assessed using a solid-phase extraction cartridge (Sep-Pak C18) and ascending instant thin-layer chromatography on silica gel (iTLC-SG) with 1 M ammonium acetate/methanol (1:1, v/v) as the mobile phase. Results were confirmed using reverse phase high-performance liquid chromatography (RP-HPLC). Radionuclide identity was verified through ^68^Ga decay analysis using an ionization chamber.^(15,16)^ The synthesis and quality control process take less than 30 min. A total of 755 [^68^Ga]Ga-DOTATATE syntheses (1,636 patient doses) were performed for clinical [^68^Ga]Ga-DOTATATE PET studies between 2014 and 2024. The system consistently achieved radiochemical yields above 89% and purities above 98%, with all quality control parameters (pH, endotoxins, filter integrity, sterility) within specifications.^(4)^

**Figure 1:**
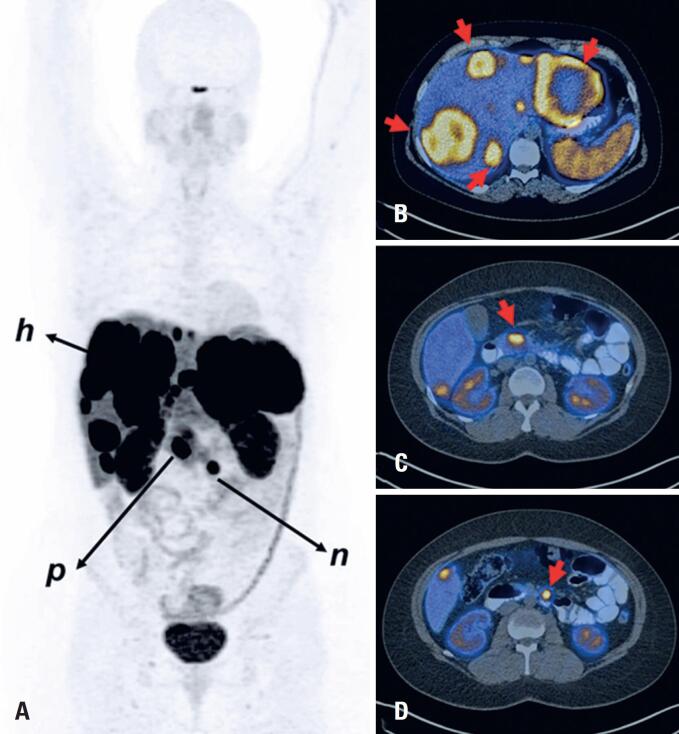
[^68^Ga]Ga-DOTATATE PET/CT imaging of a 40-year-old woman with a pancreatic neuroendocrine tumor. Whole-body PET/CT imaging was performed 60 min after intravenous radiopharmaceutical injection (235 MBq) on a Biograph mCT scanner (Siemens Medical Solutions, Germany). PET data were acquired for 4 min per bed position. Scatter correction and time-of-fly data were incorporated into the reconstruction process and low-dose CT was used for attenuation correction. (A) The maximum intensity projection (anterior view) shows foci of abnormal tracer uptake corresponding with the primary tumor in the pancreatic head (p), metastasis in a retroperitoneal paraaortic lymph node (n) and multiple liver metastases (h). (B), (C) and (D) Fused PET/CT axial slices of the abdomen show intense tracer uptake in multiple liver metastases (B), primary tumor in pancreatic head (C) and a paraaortic lymph node metastasis (D)

The routine synthesis of [^68^Ga]Ga-DOTATATE resulted in high-quality PET images for both PET-CT and PET-MR studies. Figure 1 shows a patient with pancreatic NET with lymph node and hepatic metastases as visualized using [^68^Ga]Ga-DOTATATE PET.

### [^68^Ga]Ga-PSMA-11

PSMA is a transmembrane glycoprotein that is overexpressed 100–1000 fold in 95% of prostate cancer (PCa) cells and has a low level of expression in normal tissues other than the prostate. Most PCas demonstrate PSMA expression in primary and metastatic lesions. For this reason, PSMA is considered a target for PET imaging tracers for the detection and therapy of PCa.^(17)^ PSMA inhibitor-based radiopharmaceuticals such as [^68^Ga]Ga-PSMA-11 have been successfully used to image PCa using PET.^(18)^

The most common indications for [^68^Ga]Ga-PSMA-11 PET are the localization of PCa in the setting of biochemical recurrence and primary staging of high-risk disease before surgical procedures.^(19,20)^

[^68^Ga]Ga-PSMA-11 PET demonstrated a higher diagnostic accuracy than other imaging modalities for the investigation of PCa recurrence, especially in cases of biochemical recurrence at low PSA levels.^(21,22)^ Other clinical indications for [^68^Ga]Ga-PSMA PET are described in table 1.

### [^68^Ga]Ga-PSMA-11 synthesis and quality control

The routine production of [^68^Ga]Ga-PSMA-11 was initiated in October 2015, following the standardization of the automated synthesis process using a Modular-Lab Standard module (Eckert & Ziegler) and corresponding quality control methods. A comprehensive description of this methodology was previously published by our research group.^(23)^

Radiolabelling was performed in accordance with GMP standards. Briefly, 20µg (21.12 nmol) of PSMA-11 was diluted in 1.0mL of 0.1 M NaOAc buffer at pH 4.5. Depending on the activity eluted from the ^68^Ge/^68^Ga generator, a final product activity of 1,090±35 MBq was obtained. The radiolabeling yield was >85% at a final pH of 4.5 and the radiochemical purity was >95%. Radiochemical purity was determined by ascending iTLC-SG with a mobile phase composed of 0.1 M NH^4^OAc/MeOH (1:1, v/v). These results agree with the specifications described in the European Pharmacopoeia Monography.^(4)^ Data are summarized in table 2.

**Table 2:** Mean parameters of [^68^Ga]Ga-DOTATATE, [^68^Ga]Ga-PSMA-11, [^68^Ga]Ga-DOTA-Ubiquicidin[^29-41^] and [^68^Ga]Ga-FAPi-46 synthesis

Parameters	[^68^Ga]Ga-DOTATATE	[^68^Ga]Ga-PSMA-11	[^68^Ga]Ga-DOTA-Ubiquicidin^(29-41)^	[^68^Ga]Ga-FAPi-46
Final Activity final (mCi)	20.44±4.85	25.20±4.67	14.79±3.95	18.42±3,75
Labeling Yield (%)	89.03±3.78	94.31±6.18	81.31±0.31	87.69±5.96
RCP Sep-PakC_18_ (%)	98.69±1.24	97.82±1.69	96.91±1.87	97.17±0.68
RCP TLC (%)	98.38±0.98	98.24±1.30	99.78±0.60	97.84±1.43
Pyrogen Test	negative	Negative	negative	negative
pH	4.0	4.5	4.5	4.0
Filter Integrity (Bars)	≥ 3.2	≥ 3.2	≥ 3.2	≥ 3.2
n	50	50	8	15

Values are expressed as "mean±SD".

RCP: radiochemical purity. TLC: thin layer chromatography.

From October 2015 to October 2024, 2,429 [^68^Ga]Ga-PSMA-11 syntheses (5,892 patients’ doses) were produced for clinical applications, including 4,773 doses for PET-CT (81%) and 1,119 for PET-MR (19%) for PCa diagnosis and staging/restaging.^(23,24)^

The synthesis system consistently produced [^68^Ga]Ga-PSMA-11 with high reliability and in a rapid timeframe (under 30 min), demonstrating robust and reproducible performance with high labeling efficiency, radiochemical purity, low cost, and high-quality PET imaging.

**Figure 2:**
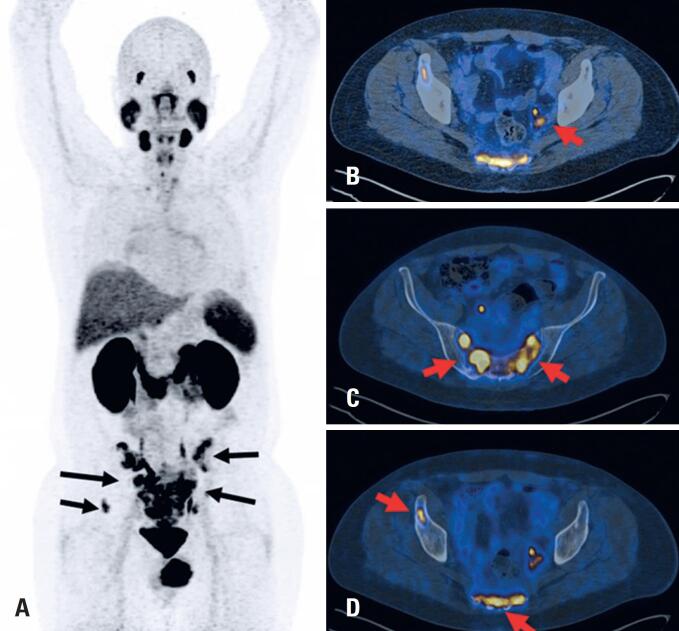
[^68^Ga]Ga-PSMA-11 PET/CT of a 62-year-old man with metastatic prostate adenocarcinoma. Whole-body PET/CT images (Biograph mCT scanner, Siemens Medical Solutions, Germany) were acquired for 4 min per bed position, 1 h after the injection of 270 MBq [^68^Ga]Ga-PSMA-11. (A) The maximum intensity projection (anterior view) shows foci of abnormal tracer uptake in multiple bone metastases in the pelvic bone. (B), (C) and (D) Fused PET/CT axial slices of the pelvis show tracer uptake in a lymph node metastasis in the left internal iliac chain (B), and bone metastases in the sacrum and right iliac bone (C and D)

Figure 2 shows a patient with metastatic PCa and secondary bone involvement detected using [^68^Ga]Ga-PSMA-11 PET.

### [^68^Ga]Ga-DOTA- Ubiquicidin_[29-41]_

Ubiquicidin_[29-41]_ is a cationic antimicrobial peptide fragment that preferentially binds to anionic bacterial cell membranes at the site of infection. Ubiquicidin_[29-41]_ is specific to several gram-positive and gram-negative bacteria and fungi. The radiolabeled fragment Ubiquicidin_[29-41]_ can differentiate between bacterial infection and aseptic inflammation. This represents an advantage over other molecular imaging methods such as [^18^F]FDG PET and scintigraphy using various tracers, including [^67^Ga]Ga-citrate, indium-111 or technetium-99m labeled leukocytes, which cannot distinguish between infection and aseptic inflammation.^(25,26)^

Initial clinical evaluation revealed that Ubiquicidin_[29-41]_ is a promising agent for imaging infectious conditions such as chronic osteomyelitis, fever of unknown origin, and suspected prosthesis infection (Table 1).^(27)^

### [^68^Ga]Ga-DOTA- Ubiquicidin_[29-41]_ synthesis and quality control

The protocol for the automated synthesis and quality control of [^68^Ga] Ga-DOTA-ubiquicidin_[29-41]_ was standardized for clinical use in the Nuclear Medicine Department in September 2021. This method has been described in detail in a previous study.^(28,29)^ Briefly, an adapted protocol template was developed and implemented for the synthesis of [^68^Ga]Ga-DOTA-Ubiquicidin_[29-41]_ using disposable GMP-grade cartridges Ubiquicidin_[29-41]_ peptide were obtained from Advanced Biochemical Compounds (ABX) (Radeberg, Germany).

[^68^Ga]GaCl_3_ was eluted from a ^68^Ge/^68^Ga generator using 0.1 M HCl. The eluate was first purified through a cationic-exchange resin and then extracted using 5.5 M NaCl/5 N HCl solution into the reaction vial, where it reacted with 50μg of DOTA- Ubiquicidin_[29-41]_ in 0.1 M NaOAc buffer at pH 4.5. Radiolabeling was performed at 95°C for 15 min. The product was purified using a SepPak C18 Plus cartridge. Radiochemical yield and purity were assessed through solid-phase extraction using a Sep-Pak C18 cartridge and ascending TLC-SG using 0.1 M sodium citrate (pH 5.5) as the mobile phase. Compound stability at room temperature was confirmed for up to 120 min by RP-HPLC, with radiochemical purity above 96%. The mean radiochemical yield and purity were greater than 80% and 96%, respectively (n=8), resulting in a sterile, pyrogen-free final product (Table 2). The [^68^Ga]Ga-DOTA-Ubiquicidin_[29-41]_ synthesis and quality control tests took less than 30 min.^(29,30)^

*In vitro* and *ex vivo* assays were used to establish the quality of the synthesized [^68^Ga] Ga-DOTA-ubiquicidin_[29-41]_ and its clinical application began in December 2021 after institutional ethics protocol approval (Research Ethics Committee of *Hospital Israelita Albert Einstein* under CAAE: 47052521.9.0000.0071). To the best of our knowledge, this is the first reported case of PET with [^68^Ga]Ga-DOTA-Ubiquicidin_[29-41]_ acquired in Brazil.^(29,30)^

**Figure 3:**
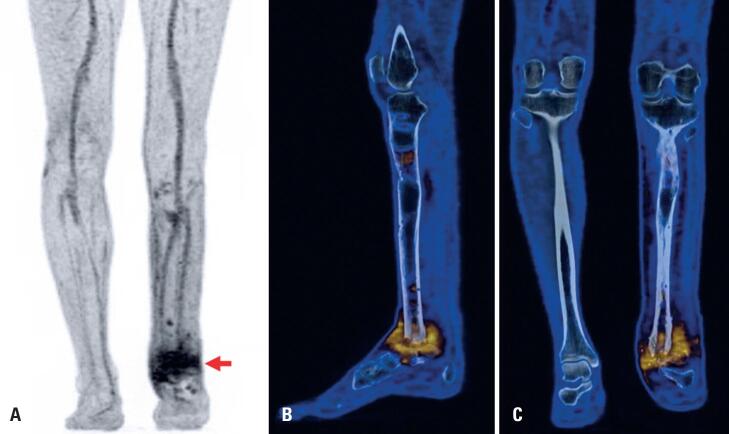
[^68^Ga]Ga-DOTA- Ubiquicidin_[29-41]_ PET/CT of a 39-year-old man with chronic osteomyelitis in the left distal tibia and ankle joint. Whole-body PET/CT imaging was performed 1 h after intravenous tracer injection (250 MBq) on a Biograph mCT scanner (Siemens Medical Solutions, Germany). PET data were acquired for 4 min per bed position. (A) The maximum intensity projection (anterior view), (B) Fused PET/CT sagittal slice of the left leg and (C) Fused PET/CT coronal slice of the legs show tracer abnormal uptake in the distal portion of the left tibia and talus and in periarticular soft tissue

Twelve patients with suspected osteomyelitis underwent PET/CT with [^68^Ga]Ga-DOTA-Ubiquicidin_[29-41]_ prior to surgery. PET/CT findings were compared with the histopathological examination and bone culture results from biopsies. [^68^Ga]Ga-DOTA-Ubiquicidin_[29-41]_ PET-CT imaging revealed specific uptake at the sites of proven infection in 11 cases. A representative case of chronic osteomyelitis identified using [^68^Ga]Ga-DOTA-Ubiquicidin_[29-41]_ PET-CT imaging is presented in figure 3.

### [^68^Ga]Ga-FAPi-46

Cancer-associated fibroblasts (CAFs) are a major subpopulation of cells in the tumor microenvironment. Fibroblast activation protein (FAP) is a membrane glycoprotein overexpressed in CAFs of several tumor types and is weakly expressed in healthy tissues. Therefore, FAP represents a promising target for both the imaging and radionuclide therapy of several tumors, especially in cancers with strong desmoplastic reactions, such as lung, pancreatic, breast, and colorectal cancers. FAP expression has been identified in more than 90% of epithelial tumors. Consequently, the development of FAP-targeting radiopharmaceuticals has garnered significant attention in recent years.^(31)^

Radiolabeled FAP inhibitors (FAPi) are currently undergoing clinical evaluation for both imaging and therapeutic applications. ^68^Ga-labeled FAPi ([^68^Ga]Ga-FAPi) has been evaluated for the diagnostic workup of several epithelial cancer types, such as pancreatic, lung, ovarian, and gastric cancers, as well as sarcomas.^(32-34)^

Many studies have demonstrated promising results for [^68^Ga]Ga-FAPi compared with those of [^18^F]FDG PET as an oncological imaging tracer. [^18^F]FDG has well-known limitations, particularly in the evaluation of tumors with low glucose avidity or metabolic activity, such as well-differentiated NET, PCa, and liver cancer.

In this context, [^68^Ga]Ga-FAPi PET/CT may be an interesting alternative for evaluating tumors with low [^18^F]FDG avidity. Moreover, the high physiological [^18^F]FDG uptake in normal organs, such as the brain, liver, and intestinal tract, decreases the sensitivity of detecting small primary or metastatic lesions in these regions. In contrast, [^68^Ga]Ga-FAPi demonstrates low uptake in normal tissues, resulting in improved image contrast and contributing to a higher sensitivity for detecting malignant lesions. Another practical advantage of [^68^Ga]Ga-FAPi is patient preparation. As FAPi uptake is independent of glycemic levels, fasting is not required, which facilitates the imaging of patients with diabetes.^(35)^ Some clinical indications for [^68^Ga]Ga-FAPi-46 PET are summarized in table 1.

### *[^68^Ga]Ga-FAPi-46* synthesis and quality control

The synthesis of [^68^Ga]Ga-FAPi-46 was implemented using disposable GMP-grade cartridges and high-purity reagents, following an adapted synthesis template with minor modifications. The synthesis procedure has been described by Fuscaldi et al.^(36)^

Briefly, [^68^Ga]GaCl^3^ was percolated through a cation-exchange resin and eluted with 0.5mL of 5.5 M HCl in saline into a reaction vial containing 50μg of FAPi-46 in 1.5mL of 0.1 M NaOAc buffer (pH=4.5) and 100µL of ethanol. The mixture was then heated at 95°C for 10 min. The resulting product was purified using a Sep-Pak C18 cartridge preconditioned with ethanol and 0.9% saline and subsequently eluted with 0.4mL of 70% ethanol. The final product was diluted with 0.9% saline and sterilized by filtration through a 0.22-µm Millipore membrane filter.

The radiochemical yield was determined by measuring the activity in the final product vial and expressing it as a percentage of the total activity, including the activity retained in the synthesis module. Radiochemical purity was assessed by ascending chromatography and Sep-Pak C18 cartridge analysis and further confirmed by RP-HPLC. Radiochemical stability was assessed using ultra-high-performance liquid chromatography. Microbiological and pyrogenicity tests, along with filter integrity testing, were performed for all batches according to the GMP guidelines. The automated synthesis yielded [^68^Ga]Ga-FAPi-46 with a mean activity of 684±67 MBq, a radiochemical yield >80%, radiochemical purity consistently exceeding 97%, and a pH of 4.5 (n=8), and remained stable above this threshold for over 120 min, indicating high radiochemical stability. Microbiological assays demonstrated that the final product was a sterile pyrogen-free solution. The filter integrity test was approved for all the batches.^(36)^

The clinical implementation of [^68^Ga]Ga-FAPi-46 PET/CT started in December 2023 after a phase III study at our institution. During this period, [^68^Ga]Ga-FAPi-46 PET/CT was performed on 31 patients. Informed consent was obtained from the participants for the publication of the images in this article.

**Figure 4:**
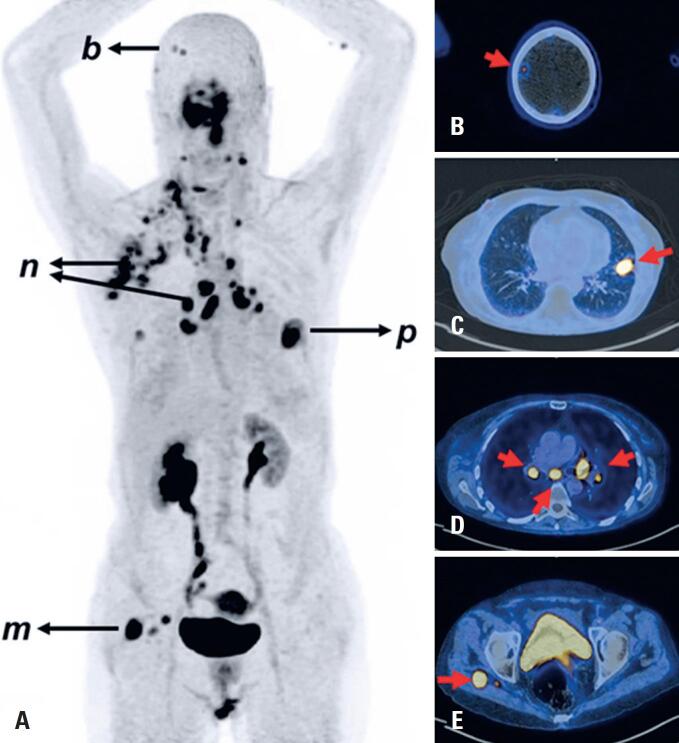
[^68^Ga]Ga-FAPi-46 PET/CT of a 63-year-old woman with non-small cell lung cancer. Whole-body PET/CT data (Biograph mCT scanner, Siemens Medical Solutions, Germany) were acquired 60 min after tracer injection (210 MBq) for 4 min per bed position and reconstructed with an iterative algorithm (A) The maximum intensity projection (anterior view) shows foci of abnormal tracer uptake corresponding with the primary tumor in the left lung (p), metastases in mediastinal, hilar, left supraclavicular, axillary and retropectoral lymph nodes (n), brain (b) and right gluteal muscle (m). (B), (C), (D) and (E) Fused PET/CT axial slices show intense tracer uptake in brain metastasis (B), primary lung tumor (C), mediastinal and hilar lymph node metastases (D), and a metastasis in the right gluteal muscle (E)

Overall, our initial experience suggests that [^68^Ga]Ga-FAPi-46 PET/CT demonstrates diagnostic performance comparable to that of [^18^F]FDG PET/CT in the tumor types evaluated.^(37)^ However, [^68^Ga]Ga-FAPi-46 PET/CT demonstrated superior lesion visualization compared to [^18^F]FDG in the brain, liver, and pleural and retroperitoneal lymph node metastases. These findings corroborate previously published data; however, further validation in larger patient cohorts is warranted to determine the diagnostic role of [^68^Ga]Ga-FAPi-46 PET/CT for different tumor types. Figure 4 shows the [^68^Ga]Ga-FAPi-46 PET-CT scan of a patient with non-small cell lung cancer, demonstrating intense tracer uptake in the primary tumor as well as in the mediastinal, hilar, neck, and axillary lymph nodes, as well as in brain and muscle metastases.

## CONCLUSION

[^68^Ga]Ga-PSMA-11 and [^68^Ga]Ga-DOTATATE are established examples of generator-based PET radiopharmaceuticals that have successful clinical applications worldwide. Emerging clinical PET agents, such as [^68^Ga]Ga-DOTA-Ubiquicidin_[29-41]_ and [^68^Ga]Ga-FAPi-46, contribute to the development of molecular imaging, promoting more precise diagnostics and improving patient care.

Over a 10-year period, the automated synthesis system for [^68^Ga]Ga-labeled radiopharmaceuticals has allowed the implementation and routine production of ^68^Ga-radiolabeled radiopharmaceuticals with high radiochemical yields and purities for PET/CT and PET/MR studies, in accordance with pharmacy practice standards and GMP conditions. During this period, more than 7.500 patients with numerous tumor types were successfully imaged at our institution. This automated system allowed the reproducible, fast, robust, and safe on-site production of PET tracers to obtain high-quality clinical PET images.
